# Accuracy of a remote quantitative image analysis in the whole slide images

**DOI:** 10.1186/1746-1596-6-S1-S20

**Published:** 2011-03-30

**Authors:** Janina Słodkowska, Tomasz Markiewicz, Bartłomiej Grala, Wojciech Kozłowski, Wielisław Papierz, Katarzyna Pleskacz, Piotr Murawski

**Affiliations:** 1 Military Institute of Medicine, Department of Pathology, Warsaw, Poland; 2 Warsaw University of Technology, Institute of Theory of Electrical Engineering, Measurement and Information System, Warsaw, Poland; 3 Medical University of Lodz, Department of Pathomorphology, Lodz, Poland; 4 Military Institute of Medicine, Department of Informatics, Warsaw, Poland

## Abstract

The rationale for choosing a remote quantitative method supporting a diagnostic decision requires some empirical studies and knowledge on scenarios including valid telepathology standards. The tumours of the central nervous system [CNS] are graded on the base of the morphological features and the Ki-67 labelling Index [Ki-67 LI]. Various methods have been applied for Ki-67 LI estimation. Recently we have introduced the Computerized Analysis of Medical Images [CAMI] software for an automated Ki-67 LI counting in the digital images.

Aims of our study was to explore the accuracy and reliability of a remote assessment of Ki-67 LI with CAMI software applied to the whole slide images [WSI].

The WSI representing CNS tumours: 18 meningiomas and 10 oligodendrogliomas were stored on the server of the Warsaw University of Technology. The digital copies of entire glass slides were created automatically by the Aperio ScanScope CS with objective 20x or 40x. Aperio's Image Scope software provided functionality for a remote viewing of WSI. The Ki-67 LI assessment was carried on within 2 out of 20 selected fields of view (objective 40x) representing the highest labelling areas in each WSI. The Ki-67 LI counting was performed by 3 various methods: 1) the manual reading in the light microscope - LM, 2) the automated counting with CAMI software on the digital images – DI , and 3) the remote quantitation on the WSIs – as WSI method. The quality of WSIs and technical efficiency of the on-line system were analysed. The comparative statistical analysis was performed for the results obtained by 3 methods of Ki-67 LI counting. The preliminary analysis showed that in 18% of WSI the results of Ki-67 LI differed from those obtained in other 2 methods of counting when the quality of the glass slides was below the standard range. The results of our investigations indicate that the remote automated Ki-67 LI analysis performed with the CAMI algorithm on the whole slide images of meningiomas and oligodendrogliomas could be successfully used as an alternative method to the manual reading as well as to the digital images quantitation with CAMI software. According to our observation a need of a remote supervision/consultation and training for the effective use of remote quantitative analysis of WSI is necessary.

## Background

The conventional hematoxylin-eosin staining is the mainstay for pathologic diagnosis, but recently immunohistochemistry has played a major role in differential diagnosis and in improving diagnostic accuracy in general surgical pathology as well as in neuro-oncologic pathology. With the increasing use of immunohistochemical [ihc] assays and the increased importance of the results, there is a need for standardization of the assays. The interpretation of ihc results may be standardized through the use of new quantitative methods.

The most relevant factor in the progression-free survival of patients with the tumours of the central nervous system [CNS] is the histological grade. An unequivocal diagnosis of the histological grade is difficult without contribution of quantitative indicators. One group of the complementary methods applied in tumours assessment is based on cell proliferation markers. Expression of the nuclear antigen Ki-67 (MIB-1) has been linked to proliferative activity and prognosis in a variety of neoplasms. Ki-67 labelling index [LI] has been used as a complementary method to differentiate better and worse prognostic groups of astrocytomas, meningiomas and oligodendrogliomas [1-4] However, Ki-67 LI reproducibility has been doubted because of the interlaboratory and interobserver variability, imprecision and low sensitivity of human visual inspection. Important possible sources of variability of the Ki-67 LI are the immunohistochemical staining methods and the counting methods [1,3-7].

In this study, we explored the accuracy of a remote Ki-67 LI quantisation performed on the whole slides images [WSI] of the CNS tumours, by the software CAMI [the Computerized Analysis of Medical Images] installed as a Web-based application along with networked server [9].

## Methods

The study was performed on two types of CNS tumours: oligodendrogliomas and meningiomas selected from the archives of the Pathology Department of the Military Institute of Medicine, and of the Pathomorphology Department of the Medical University of Lodz.

For the Ki-67 LI evaluation two various methods of ihc staining were applied for two groups of samples A and B as follows:

**Group A**. 28 representative samples of tumours: 10 oligodendrogliomas and 18 meningiomas graded I - III were stained according to the standard manual ihc procedure for Ki-67 nuclear antigen on paraffin sections, with the monoclonal mouse anti-human Ki-67 antibody (clone MIB-1; manufacturer Dako, Denmark; product no. M7240), in dilution of 1:100. Dako's EnVisionTM (product no. K4001) was used as the visualisation system. The insufficient quality of 8 glass slides stained ihc (scanty vs. fragmented or fibrotic samples vs. overstained, or thick multilayer samples) were the problems for the digital images acquisition and scanning (focusing and calibration) followed by the difficulties in quantitative assessment of MIB-1 reactivity. Finally these cases were excluded from the further investigation. In order to prove the quality standard of glass slides the procedure of ihc staining was modify and repeated in the group B.

**Group B.** The paraffin blocks representing 20 cases of group A were re-cut for 3 μm thick sections and autostained (in Dako’s autostainer) with the FLEX monoclonal mouse anti-human Ki-67 antigen, clone MIB-1, ready-to-use, according to the manufacturer’s manual [8].

Three techniques of Ki-67 LI quantitation were performed by the pathologists for the group A and B samples:

a/ LM method - the labelled-cell count (Ki-67 LI) was determined by a manual reading with the light microscope. The number of tumour cells with distinct nuclear staining was recorded after counting tumour cells in 2 consecutive high power fields in the most reactive areas of the slides. The percentage of positive tumour cells was then calculated as Ki-67 labelling index.

b/ DI method - the automated counting on the digital images with CAMI software. This method based on Ki-67 LI quantitation in 2 out of 20 recorded digital images representing 20 high power fields with the highest MIB-1 reactivity. For the digital images acquisition 20 high power microscopic fields (40x) with the highest MIB-1 immunoreactivity were selected from each slide. The selection was performed with the Olympus BX-61 microscope at 400x magnification. The Olympus DP72 digital camera working in red-green-blue (RGB) format and resolution 680 × 512 was used for the images preparation. Total number of 370 digital images underwent the investigation. Each digital image covered field of 320 × 240 µm with the mean number of neoplastic cells as 596 (ranging from 132 to 967). The following procedure was the images analysis based on the recently developed computerised automated system for cell counting - CAMI [5,9].

c/ WSI method - the automated counting with CAMI algorithm on the whole slide images. Each glass slide was automatically converted to the WSI using the Aperio ScanScope SC (Aperio Technologies, U.S.A.) under 20x or 40x for the group A and only under 40x for the group B. The WSI were stored on the server of the Institute of Theory of Electrical Engineering (http://cami.iem.pw.edu.pl) The server speed connection for downloading was 34 Mb/s and for uploading 15 Mb/s [10]. The Aperio’s Image Scope software was used for a remote screening of the WSIs and selection of 20 high power fields (40x) with the highest MIB-1 immunoreactivity - by the pathologist at the workstation of the MIM (speed connection for downloading 28 Mb/s and for uploading 15 Mb/s). The recorded 20 images (snapshots) of each case were evaluated with CAMI algorithm for Ki-67.

For the method DI and WSI the final Ki-67 LI results were calculated on the base of 2 out of 20 the highest values among recorded results for each case as follows:

Ki-67 = ((R_1_+R_2_)/(N_1_+R_1_+N_2_+R_2_)) x100% [%]

N_1_ – number of cells with negative immunoreaction for area A; R_1_ – number of cells with nuclear reactivity for area A; N_2_ – number of cells with negative immunoreaction for area B; R_2_ – number of cells with nuclear reactivity for area B.

The comparative statistical analysis of the results was performed with the Wilcoxon’s test.

## Results

**Figure 1:**
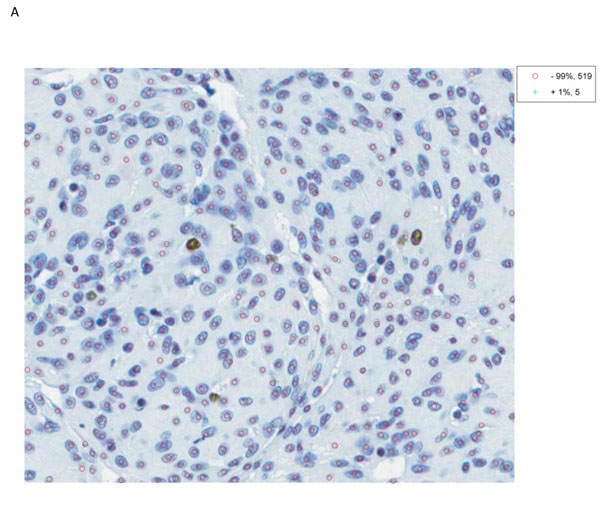
**MIB-1 immunohistochemical staining in selected tumours.A. Meningothelial meningioma.** The digital image of the microscopic field showing scanty immunoreactive cells (brown nuclei marked with “+”). Low proliferative activity (Ki-67 LI - 1%) corresponds with the histological tumour grade I. **B. Anaplastic oligodendroglioma.** The digital image presenting numerous immunoreactive tumour cells assessed with the CAMI software as 21,5% (Ki-67 LI) which support the diagnosis of anaplastic (grade III) oligodendroglioma.

An exemplary figure of the obtained digitized slides is given in figure 1A, 1B. The histopathological characteristics of the studied CNS tumours is presented in the table 1.

**Table 1:** The histopathological characteristics of the studied material

Histological type of tumour	Histological Grade	Group A		Group B
		
		Number of cases		Number of cases
		
		Preliminary selection	Final selection	Final selection
Oligodendroglioma	II	4	0	0
Oligodendroglioma	III	6	4	4
Meningioma	I	8	7	7
Meningioma	II	7	7	7
Meningioma	III	3	2	2
Total		28	20	20

All histological ihc samples, the digital images and the WSIs of the group B showed prefect quality, much better than those of the group A. The glass slides of the group A in majority (15/20) were scanned under 20x but for the standard protocol of the study the WSIs were screened and counted under 40x. The simulated higher magnification decreased the images quality on a monitor, impaired the computerised image analysis of DIs and WSIs and could be a reason of some discrepancies between counting results in a few cases.

The final data of Ki-67 LI evaluation listed according to 3 modes of quantisation: the manual reading - LM, and the automated assessment in the digital images – DI and in the whole slide images – WSI method are shown in the table 2.

**Table 2:** Results of Ki-67 LI counted by various methods, in relation to the histological type of tumours and different methods of ihc staining (group A and B)

The histological groups of tumours	Ki-67 results (%)					
	
	Methods of counting					
	
	Group A			Group B		
**Oligodendroglioma III**	LM	DI	WSI	LM	DI	WSI
1.	17,10	17,35	27,20	19,30	19,20	25,80
2.	28,50	29,45	27,15	31,30	29,15	33,10
3.	41,40	41,85	36,15	74,20	76,90	46,20
4.	25,20	24,75	31,55	22,90	22,45	16,95
**Meningioma I**						
1.	1,85	2,05	2,00	2,40	1,90	2,55
2.	8,95	10,00	11,79	10,30	8,50	8,60
3.	6,50	5,75	7,10	4,25	4,40	6,35
4.	5,30	5,60	5,85	6,45	6,80	6,25
5.	8,65	9,60	6,60	4,70	4,75	4,05
6.	5,95	6,80	8,32	10,90	9,70	16,10
7.	7,60	7,90	9,55	15,95	15,60	23,55
**Meningioma II**						
1.	19,20	20,15	19,80	7,30	7,70	6,20
2.	5,90	8,55	5,61	16,20	16,35	16,60
3.	9,95	9,20	11,30	11,00	11,20	15,90
4.	18,60	19,30	17,85	11,80	12,80	12,60
5.	8,85	8,70	15,60	13,90	14,05	20,75
6.	20,90	24,95	26,00	32,60	33,40	35,70
7.	21,30	21,25	20,90	14,50	15,40	17,00
**Meningioma III**						
1.	23,35	21,90	10,10	32,60	35,10	27,25
2.	25,60	26,95	22,50	23,65	23,80	32,75

Legend: the results are presented as a weighted average from 2 view fields showing the highest MIB-1 immunoreactivity (Ki-67).

As presented in the table 2 the average weight values of Ki-67 obtained by the WSI method are higher than parallel values of the LM and DI methods in both groups: A and B. Additionally it is shown that the average weight values of Ki-67 resulted from WSI method in the group B are higher than those in the group A. These observations can suggest the greater sensitivity of the WSI method comparing to the methods LM and DI but need to be proved in the extended study.

The comparative analysis of statistical distributions of variables (values of Ki-67) for the methods LM, DI, WSI in the groups A and B as well as the groups A vs. B was carried on by the Wilcoxon’s test. Due to statistical character of the variables the null hypothesis (H_0_) was proposed that the distributions of both variables are identical by means that the results of the methods correspond to the medical means of the results. As a result of statistical analysis we didn’t find a reason to reject the null hypothesis. This is why, in our opinion, the WSI method could be used as an alternative one to the LM and DI counting methods in both the group A and group B. Exceptionally, in one case (1/15) in the population of “Meningioma II” the statistical significance was lower than 0,05 (p<0,02) (Table 3) which indicates that the methods LM-B vs. DI-B shouldn’t to be used interchangeably.

**Table 3:** The statistical results (probability of value that H_0_ should be rejected) of the comparative analysis for three counting methods for Meningiomas II.

	DI - A	LM - A	WSI - A	DI - B	LM - B	WSI - B
**DI - A**		0,24	0,87	1,00	1,00	0,55
**LM - A**	0,24		0,24	0,87	1,00	0,50
**WSI - A**	0,87	0,24		0,61	0,61	0,74
**DI - B**	1,00	0,87	0,61		**0,02**	0,09
**LM - B**	1,00	1,00	0,61	**0,02**		0,06
**WSI - B**	0,55	0,50	0,74	0,09	0,06	

Legend: The probability of value in the table 3 could be in range 0-1. For the underline value null hypothesis H_0_ must be rejected.

## Discussion

Recent progress in the pathology field is headed towards a more quantitative, reproducible, and specific approaches. Image analysis with new tools and newer stains allow for shifting from semiquantitative to more quantitative analyses in some areas of the pathology. The conventional hematoxylin-eosin staining is the mainstay for pathologic diagnosis, but immunohistochemistry has played a major role in differential diagnosis and in improving diagnostic accuracy in general surgical pathology as well as in neuro-oncologic pathology. With the increasing use of ihc assays and the increased importance of the results, there is a need for standardization of the assay. The interpretation of ihc results may be standardized through the use of newer stains (e.g. MIB-1 vs. MIB-1Flex antibody; autostainers) and tools such as WSI technology and new quantitative methods. With the assistance of a computer and automated analysis programs some help is offered to eliminate the inherent variability of pathologist-based scoring and to increase the sensitivity of the quantitative trials.

It has been demonstrated that the Ki-67 proliferation index is the most important criterion for histological classification of meningiomas, oligodendrogliomas or astrocytomas [1,3,4]. The computer-aided image analysis software CAMI has been designed and implemented in our previous research on Ki-67 LI on the CNS tumours [5,9]. In the current study CAMI was installed as a Web-based application along with networked server for the remote quantitate analysis of Ki-67 LI on the whole slide images of meningiomas and oligodnedrogliomas. The accuracy of the remote automated quantisation of Ki-67 LI of the WSIs was evaluated by the comparative quantitative study with two other methods of counting: the manual reading with the traditional microscope and with the direct automated image analysis by CAMI algorithm. The statistical analysis of the counting data showed that the WSI method could be used as an alternative one to both mentioned methods: the LM and DI counting. The higher values of Ki-67 LI were noticed for the WSI method in comparison to the LM and DI methods, regardless of a mode of immunohistochemical staining (the groups A and B). Such results might suggest the greater sensitivity of the WSI method in comparison to the others however it needs extended study to confirm it. The interpretation of the values of Ki-67 LI higher in the group B than in the group A for the WSI method is not unequivocal in this model of study. This might be related to the various standards of the immunohistochemical procedures in the group A and B, the different magnification used for the scanning process, and also to a human factor in the counting fields selection.

## Conclusions

The results of our investigations indicate that the remote automated Ki-67 LI analysis performed with the CAMI algorithm on the whole slide images of meningiomas and oligodendrogliomas could be successfully used as an alternative method to the manual reading as well as to the direct automated image quantitation with CAMI software.

Our data also suggest the greater sensitivity of the WSI method comparing to other used methods of Ki-67 LI assessment however this statement needs more extended study to confirm it. The presented results will be the subject of further research to verify the submitted proposals on the numerically larger groups so that it would make possible to draw conclusions about a population of CNS tumours.

## List of abbreviations

ihc: immunohistochemical; CNS: the central nervous system; Ki-67 LI: Ki-67 labelling index; WSI: the whole slide image; CAMI: the Computerized Analysis of Medical Images; LM: light microscopy; DI: digital image

## Competing interests

The authors declare that they have no competing interests.
